# Impact of Resection Margins and Adjuvant Therapy on Survival Outcomes in Lymph Node-Negative Distal Cholangiocarcinoma

**DOI:** 10.3390/curroncol32030178

**Published:** 2025-03-19

**Authors:** Hye Jin Kang, In Young Jo

**Affiliations:** 1 Department of Radiation Oncology, Incheon St. Mary’s Hospital, College of Medicine, The Catholic University of Korea, Seoul 06591, Republic of Korea; kanghj@catholic.ac.kr; 2 Department of Radiation Oncology, College of Medicine, Soonchunhyang University Cheonan Hospital, Cheonan 31151, Republic of Korea

**Keywords:** distal cholangiocarcinoma, resection margin, adjuvant therapy, treatment outcome, prognostic factor

## Abstract

The prognostic value of the resection margin (RM) status and the efficacy of adjuvant therapy (AT) in distal cholangiocarcinoma (CCC) are unclear. RM status appears particularly impactful in lymph node-negative distal CCC, representing early-stage disease. The prognostic value of RM status was investigated, and subpopulations of patients with lymph node-negative distal CCC who might benefit from AT were identified. Overall, 139 patients with distal CCC who underwent surgical resection between March 2006 and December 2023 were analyzed. RM status was categorized as wide (>5 mm) in 65 patients (46.8%), close (≤5 mm) in 32 patients (23.0%), or positive in 42 patients (30.2%). AT was administered to 48 patients (34.5%). Patients with close or positive RMs achieved significantly lower locoregional control (LRC) than those with wide RMs. However, overall survival (OS) did not differ across the three RM groups. The impact of RM status was more evident in patients not receiving AT. Patients with wide RMs exhibited better 3-year LRC, progression-free survival (PFS), and OS rates (79.0%, 66.5%, and 69.1%, respectively) than those with close (21.7%, 15.7%, and 34.4%) or positive RMs (44.3%, 25.3%, and 50.2%, respectively). No significant differences were found between close and positive RM groups. AT appears to have improved LRC and PFS in patients with close or positive RMs but not in those with wide RMs. Close RMs were associated with poor outcomes comparable to those with positive RMs. These results indicate that achieving adequate RM width is crucial for improving survival. Moreover, AT may improve survival when adequate RMs cannot be achieved. Nonetheless, larger studies are needed to validate these findings.

## 1. Introduction

Cholangiocarcinoma (CCC) is a highly heterogeneous group of malignant neoplasms originating from the bile ducts [1]. Distal CCC, which arises in the segment between the cystic duct and the ampulla of Vater, constitutes 20–30% of all CCC cases [1,2]. Surgical resection is the only curative treatment for distal CCC [3,4]. However, prognosis after resection is poor, with 5-year overall survival (OS) rates ranging from 20% to 40% [5,6].

Adjuvant therapy (AT) has been explored as a strategy to improve survival, but its efficacy and appropriate indications remain uncertain [7]. Current guidelines recommend AT for high-risk patients based on the assessment of lymph node involvement and resection margin (RM) status [8]. Lymph node involvement is a well-established major risk factor for poor survival [9,10,11], with increasing lymph node metastases correlating with worse prognosis [10]. However, the prognostic value of RM status in patients with distal CCC remains unclear. Although positive RMs are generally associated with unfavorable outcomes [12,13,14,15], a recent study found no significant survival differences between RM statuses [16]. Nonetheless, the survival benefit of negative RM status may be limited to lymph node-negative patients [17,18,19]. This observation suggests that the impact of RM status on treatment outcomes is more strongly pronounced in patients with lymph node-negative distal CCC who are typically in the earlier stages of the disease. Moreover, the potential survival benefit of achieving an optimal RM width beyond securing a negative RM is unclear.

This study evaluated the prognostic value of RM status in patients with lymph node-negative distal CCC and identified specific patient subgroups who might benefit from AT.

## 2. Materials and Methods

### 2.1. Patient Selection

A total of 289 consecutive patients with distal CCC who underwent curative-intent surgical intervention at two institutions between March 2006 and December 2023 were retrospectively evaluated. All patients underwent physical examinations, preoperative tumor marker testing for the carcinoembryonic antigen (CEA) and carbohydrate antigen 19-9 (CA 19-9), and imaging evaluations. Tumor extension and resectability were assessed utilizing dynamic computed tomography (CT) and/or dynamic magnetic resonance imaging (MRI). Biliary anatomy and the extent of obstruction were evaluated using endoscopic or percutaneous cholangiography. Patients with obstructive jaundice or bile duct dilatation underwent preoperative biliary drainage.

The exclusion criteria were as follows: (1) patients preoperatively suspected of having distal CCC but subsequently pathologically diagnosed with pancreatic cancer; (2) those who did not achieve complete macroscopic resection; (3) patients with a follow-up duration of less than 3 months following surgery; and (4) those who did not undergo lymph node dissection or had confirmed lymph node involvement. Of the 289 patients, 139 were included in the study. This study was approved by the Institutional Review Boards of Incheon St. Mary’s Hospital, The Catholic University of Korea (reference number: OC25RADI0003), and Soonchunhyang University Cheonan Hospital (reference number: SCHCA 2024-07-047). All data were retrieved from the medical reports and institutional medical records. The need for informed consent was waived because of the study’s retrospective nature.

### 2.2. Treatment and Pathological Evaluation

A total of 103 patients (74.1%) underwent pancreaticoduodenectomy, and 36 patients (25.9%) underwent bile duct resection alone. All patients underwent regional lymph node dissection. The portal vein was resected and reconstructed in cases of suspected portal-vein invasion. Tumor staging was determined based on the eighth edition of the American Joint Committee on Cancer staging system [20].

Positive RMs were defined as the presence of high-grade dysplasia, in situ carcinoma, or invasive carcinoma at the edge of the specimen. Intraoperative frozen section analysis was routinely performed on proximal and/or distal bile duct margin segments excised from the specimen. If the bile duct RM was positive on frozen section analysis, an additional resection was performed when feasible to achieve a negative RM. Among 50 patients with positive ductal RMs confirmed through frozen section analysis, 33 underwent additional resection. Of these, 14 retained positive RMs, and 19 achieved clear RMs. Ductal RM status was reclassified after the frozen sections and formalin-fixed slides of the resected margin were reviewed. Eight patients initially identified as having negative ductal RMs during frozen-slide analysis were subsequently found to have positive ductal RMs when formalin-fixed slides were reviewed. The presence of cancer cells at the radial margin on formalin-fixed slides was also classified as positive RM. Five patients had positive radial margins, including two with positive ductal and radial RMs. Among the 97 patients with clear RMs, the RMs were subdivided into wide and close categories. Margins were classified as close if cancer cells were ≤5 mm from the cut surface and wide if cancer cells were >5 mm away, based on a previous study that established a cutoff of 5 mm as a clinically significant threshold for CCC [21]. Ultimately, the RM status was categorized as wide in 65 patients (46.8%), close in 32 (23.0%), and positive in 42 (30.2%).

Forty-eight patients (34.5%) received AT after surgical resection, and 29 underwent adjuvant chemotherapy. The chemotherapy regimens included capecitabine in 23 patients (79.4%), 5-fluorouracil (5-FU) plus leucovorin in four (13.8%), 5-FU plus cisplatin in one (3.4%), and docetaxel plus cisplatin in one (3.4%). The remaining 19 patients, most with positive RMs (84.2%), were treated with concurrent chemoradiotherapy (CCRT), including 5-FU plus leucovorin in 11 patients (57.9%) and capecitabine in eight (42.1%). Radiotherapy (RT) was administered to the tumor bed alone in nine patients (47.4%) and to the tumor bed and regional lymphatics in 10 patients (52.6%). The median RT dose was 50.4 Gy (range: 45–60 Gy) at 1.8–2.0 Gy per fraction. All patients were treated with intensity-modulated RT.

### 2.3. Follow-Up and Statistical Analysis

In most cases, the first follow-up was conducted 1 month after treatment. Subsequently, patients were followed up with every 3 to 6 months for the first 2 years, every 6 months until 5 years, and annually thereafter. The standard follow-up protocol included a clinical history review, physical examinations, imaging evaluations, and laboratory analyses, including CEA and CA 19-9 tests. Radiographic imaging was performed using dynamic CT, and dynamic MRI or positron emission tomography was performed as supplementary modalities for detailed assessment of potential recurrence.

Between-group comparisons were performed using the chi-square test. The Kaplan–Meier method was employed to estimate locoregional control (LRC), progression-free survival (PFS), and OS. Pairwise differences in survival were analyzed using the log-rank test. LRC was defined as the time from the date of surgical resection to either locoregional recurrence or the last follow-up visit in disease-free patients. Locoregional recurrence was defined as recurrence at the anastomosis site or along the surgical bed near the superior mesenteric or hepatic arteries. PFS was defined as the time from surgical resection to the first recurrence or last follow-up visit. OS was defined as the time from surgical resection to death from any cause or the last follow-up visit.

Prognostic factors associated with survival outcomes were identified using a univariate Cox proportional hazards model. Potential prognostic factors (*p* < 0.100) in the univariate analysis were included in a multivariate Cox proportional hazards model. All tests were two-sided. Statistical significance was set to *p* < 0.050. All statistical analyses were performed using R software, version 4.3.2 (R Foundation for Statistical Computing, Vienna, Austria).

## 3. Results

### 3.1. Patient Characteristics

Patient characteristics are summarized in Table 1. The median age at the time of surgery was 70 years (range, 31–85 years), with a nearly equal male-to-female ratio. Preoperative elevated CEA (>5 ng/mL) and CA 19-9 (>37 U/mL) levels were identified in 12 (8.6%) and 107 (77.0%) patients, respectively. The median tumor size was 25 mm (range, 4–75 mm). Portal-vein resection was performed in five patients (3.6%). Lymphovascular invasion was observed in 44 patients (31.7%), and perineural invasion was identified in 91 patients (65.5%). Tumors in 35 patients (25.2%) were classified as T1. All patients underwent lymph node dissection, with an average of 13 dissected lymph nodes. Bile duct resection alone was frequently performed in the group with positive RMs. The incidence of lymphovascular invasion was significantly higher, and the number of dissected lymph nodes was significantly lower in this group. No other significant differences in patient characteristics were observed among the three groups.

### 3.2. Survival Outcome According to the RM Status

The median follow-up period was 33 months (range: 7–151 months). The median time to recurrence among the 73 patients (52.5%) who recurred was 33 months. This identical period reflects the distribution of recurrence events in our cohort, with the wide follow-up range (7–151 months) ensuring robust survival estimates via Kaplan–Meier analysis. Log-rank tests confirmed significant differences in survival outcomes, increasing the reliability of our findings. The initial recurrence patterns were isolated local recurrence in 35 patients (25.2%), isolated distant recurrence in 22 patients (15.8%), and concomitant local and distant recurrence in 16 patients (11.5%). Local recurrence was observed in 20 of 65 patients (30.8%) with wide RM, 14 of 32 (43.8%) with close RM, and 17 of 42 (40.5%) with positive RM. Distant recurrence was observed in 16 of 65 patients (24.6%) with wide RM, 8 of 32 (25.0%) with close RM, and 14 of 42 (33.3%) with positive RM. The distribution of initial recurrence patterns did not differ significantly according to RM status (local recurrence, *p* = 0.382; distant recurrence, *p* = 0.580). The most common sites of distant recurrence were the liver (23 patients), peritoneum (six patients), lungs (five patients), and multiple organs (four patients).

**Figure 1:**
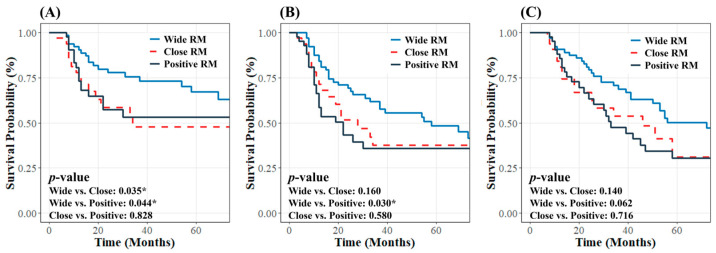
Kaplan–Meier curves for (**A**) locoregional control, (**B**) progression-free survival, and (**C**) overall survival according to resection margin status in the entire patient cohort. * indicates statistical significance (*p* < 0.05).

The 3-year LRC and PFS rates for all patients were 62.6% and 48.8%, respectively. Regarding RM status, the 3-year LRC rates were 75.6% for wide RM, 47.8% for close RM, and 53.1% for positive RM, with significantly lower rates for close and positive RMs than for wide RM (*p* = 0.035 and *p* = 0.044, respectively). The 3-year PFS rates were 61.7%, 37.5%, and 36% for wide, close, and positive RMs, respectively. The difference was significant between wide and positive RM status (*p* = 0.030) but not between wide and close RM status (*p* = 0.160) (Figure 1A,B).

During follow-up, 75 patients (54%) died, including 63 cancer-related deaths and 12 deaths from other causes, with a median OS of 51 months. The 3-year OS rate in the entire cohort was 59.2%. The 3-year OS for wide RM was similar to that for close RM (68.8% vs. 53.6%, *p* = 0.140) and positive RM (68.8% vs. 47.5%, *p* = 0.062) (Figure 1C).

### 3.3. Association Between Survival Outcomes and AT

**Figure 2:**
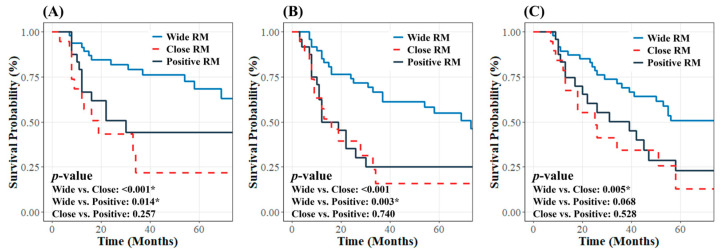
Kaplan–Meier curves for (**A**) locoregional control, (**B**) progression-free survival, and (**C**) overall survival according to resection margin status in patients who did not receive adjuvant therapy. * indicates statistical significance (*p* < 0.05).

Among 139 patients, 91 (65.5%) underwent surgical resection alone without AT. The subgroup analysis revealed that differences in survival outcomes based on RM status were more evident among subgroups than in the total cohort. Patients with wide RMs demonstrated significantly better 3-year LRC, PFS, and OS rates (79.0%, 66.5%, and 69.1%, respectively). In contrast, patients with close RMs had lower rates (21.7%, 15.7%, and 34.4%; *p* < 0.001, *p* < 0.001, and *p* = 0.005, respectively). Similarly, patients with positive RMs exhibited 3-year LRC, PFS, and OS rates of 44.3%, 25.3%, and 50.2%, respectively (*p* = 0.014, *p* = 0.003, and *p* = 0.068). In the cohort that did not undergo AT, patients with close RMs had lower LRC, PFS, and OS than those with positive RMs, though differences were not significant (*p* = 0.257, *p* = 0.740, *p* = 0.528). This likely reflects variability due to the small sample size (19 and 24 patients) (Figure 2).

**Figure 3:**
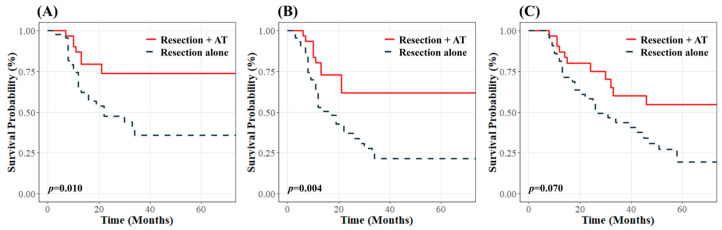
Kaplan–Meier curves for (**A**) locoregional control, (**B**) progression-free survival, and (**C**) overall survival according to the use of adjuvant therapy in patients with close and positive resection margins.

In the total cohort, AT did not effectively improve 3-year LRC (70.4% vs. 59.2%, *p* = 0.200), PFS (55.7% vs. 45.7%, *p* = 0.300), and OS (62.6% vs. 57.4%, *p* = 0.500). Among patients with wide RM, 17 (26.2%) received AT, whereas 48 (73.8%) did not. AT did not improve 3-year LRC (66.3% vs. 79.0%, *p* = 0.600), PFS (47.6% vs. 66.5%, *p* = 0.100), or OS (68.4% vs. 69.1%, *p* = 0.500) in this group. In patients with close or positive RMs, 31 (41.9%) received AT, and 43 (58.1%) did not. AT was associated with better 3-year LRC, PFS, and OS than the absence of AT (73.9%, 61.7%, and 60.0% vs. 35.9%, 21.6%, and 19.5%; *p* = 0.010, *p* = 0.004, and *p* = 0.070, respectively) (Figure 3). In the group with close RMs, AT tended to improve 3-year LRC (80.8% vs. 21.7%, *p* = 0.006), PFS (69.2% vs. 15.7%, *p* = 0.005), and OS (84.6% vs. 34.4%, *p* = 0.020), although these findings should be interpreted with caution because of the small number of patients in this group.

### 3.4. Prognostic Factors

To identify the prognostic factors associated with treatment outcomes, the following parameters were analyzed: age (<70 vs. ≥70 years), CEA levels (≤5 vs. >5 ng/mL), CA 19-9 levels (≤37 vs. >37 U/mL), surgical procedure (pancreaticoduodenectomy vs. bile duct resection), tumor size (<3 vs. ≥3 cm), histologic grade (well/moderately differentiated vs. poorly differentiated), portal-vein invasion (absent vs. present), pancreatic invasion (absent vs. present), duodenal invasion (absent vs. present), lymphovascular invasion (absent vs. present), perineural invasion (absent vs. present), T stage (1 vs. 2–3), and the use of AT (no vs. yes). The results of the multivariate analysis of the prognostic factors are presented in Table 2. Positive RM predicted worse LRC (hazard ratio [HR], 1.950; *p* = 0.048) and PFS (HR, 1.898; *p* = 0.019), whereas close RM was significantly associated with worse LRC (HR, 2.308; *p* = 0.023). Elevated CA 19-9 levels strongly predicted poor LRC (HR, 3.164; *p* = 0.011) and PFS (HR, 2.265; *p* = 0.016). An advanced T stage correlated with poor LRC (HR, 1.039; *p* = 0.044). Lymphovascular invasion was significantly associated with poor PFS in the univariate analysis but not in the multivariate analysis. Lymphovascular invasion was the only prognostic factor significantly associated with worse OS (HR, 2.319; *p* < 0.001).

## 4. Discussion

This study evaluated the survival outcomes of patients with lymph node-negative resected distal CCC, focusing on the prognostic impact of RM status. Patients with wide RM exhibited significantly better LRC than those with close or positive RM and better PFS than those with positive RM, and this effect was more evident in the subgroup that underwent surgical resection without AT. Wide RMs were associated with notably better LRC, PFS, and OS than close and positive RMs, although OS improvement over positive RMs did not reach statistical significance. Additionally, close RMs showed no significant survival advantage over positive RMs, underscoring the need to achieve an optimal RM width beyond negativity. Positive RMs are associated with higher recurrence and poorer prognosis, aligning with our findings [12,13,14,15,22]. For instance, Im et al. demonstrated that patients with negative RMs had significantly better 3-year LRC (56.5% vs. 15.9%, *p* < 0.001) and OS (60.2% vs. 14.3%, *p* < 0.001) than those with positive RMs [22]. Nevertheless, the impact of close RMs on treatment outcomes is unclear despite our observation that close and positive RMs had similar effects. Kim et al. found that a 10 mm cutoff distinguished wide RMs with superior 5-year disease-specific survival from close RMs in extrahepatic CCC (60.4% vs. 42.8%, *p* = 0.004) [23]. A prospective study defined close RM in distal CCC as ≤1 mm. Patients with close RM exhibited 5-year PFS and OS rates of 14.9% and 22.3%, respectively, similar to those with positive RM (PFS: 14.4%, OS: 21.8%) but significantly worse than patients with wide RM (PFS: 39.2%, *p* < 0.001; OS: 52.2%, *p* < 0.001) [24]. These findings demonstrate that RM status is a cornerstone of prognosis. Nonetheless, other risk factors affect outcomes.

Beyond RM status, we identified additional prognostic factors associated with survival outcomes. Preoperative elevated CA 19-9 levels were a good predictor of worse LRC and PFS, consistent with studies linking high CA 19-9 to adverse outcomes [25,26]. Yamamoto et al. showed that preoperative CA 19-9 reflected systemic disease burden, and negative RMs improved 5-year OS only in patients with normal CA 19-9 levels [27]. Similarly, the T stage, reflecting tumor invasion depth, significantly influenced LRC, but not PFS and OS, in multivariate analysis, aligning with a previous study showing that the T stage was associated with broader risk factors, including lymph node metastasis and perineural invasion [28]. Lymphovascular invasion was a good predictor of OS and early recurrence within 1 year, consistent with the literature [29]. These factors enhance our understanding of prognosis in distal CCC.

AT can potentially improve prognosis; however, its benefits and indications have yet to be clarified. In our study, AT did not improve survival outcomes across the cohort but showed a trend toward enhancing LRC and PFS in the close and positive RM subgroups, with a suggestion of better OS. In contrast, AT did not increase survival in patients with wide RMs, suggesting that AT is more effective in patients with suboptimal RMs. Despite the small subgroup sizes in these exploratory analyses, the apparent survival advantage in close RM patients suggests AT may benefit high-risk cases, though findings remain preliminary. A meta-analysis found that AT improved OS in patients with lymph node-positive disease (odds ratio [OR]: 0.49, *p* = 0.004) and those with positive RMs (OR: 0.36, *p* = 0.002) but not in the overall cohort [30]. The BILCAP trial reported that adjuvant capecitabine improved survival in a per-protocol analysis (HR: 0.75, *p* = 0.028) [31]. Additionally, the SWOG S0809 trial demonstrated comparable 2-year PFS (54% vs. 48%) and OS (67% vs. 60%) between negative and positive RM subgroups, with the improvement in treatment outcomes for positive RM patients—reaching survival rates similar to those with negative RMs—attributed to the beneficial effects of CCRT [32]. Current guidelines, reflecting such evidence, recommend AT with capecitabine for high-risk patients, particularly those with lymph node involvement and a positive RM status, and recommend the use of CCRT in positive RM cases [8,33]. These results indicate the potential efficacy of AT in high-risk groups, and patients with close RMs may benefit from AT.

This study involved limitations. First, selection bias may have affected the AT use and modality choice. AT was less frequent in patients with wide RMs, likely due to better prognoses, and more frequent in those with close or positive RMs. This included CCRT for positive RMs (to reduce locoregional recurrence risk) and chemotherapy alone in other cases. This heterogeneity in AT modalities aligns with clinical practice and does not weaken our findings, which focus on AT’s broader impact across RM statuses. Nonetheless, the lack of effect of AT in patients with wide RMs should be interpreted with caution because the lower frequency of use may obscure efficacy. The variable inclusion of CCRT further complicates comparisons across groups. Second, patients were followed up with for more than 10 years, during which time AT practices evolved considerably, and several patients treated before capecitabine and 5-FU standardization received different chemotherapy regimens, adding treatment heterogeneity; however, most regimens included capecitabine or 5-FU. Third, the small number of patients, especially in subgroup analyses, limits statistical power and generalizability, potentially affecting the robustness of the results in the subgroups. Nonetheless, Kaplan–Meier analyses and log-rank tests yielded consistent survival estimates across RM groups, strengthening the reliability of our findings. Additional randomized controlled trials with larger cohorts, longer follow-up periods, and standardized AT protocols are needed to validate these exploratory findings.

## 5. Conclusions

In lymph node-negative distal CCC, an early-stage disease, RM status is critical in determining patient prognosis. Securing an optimal RM width beyond a negative margin is essential, as close RMs show survival outcomes similar to positive RMs—a novel finding not widely recognized in prior research. For patients with close or positive RMs, where wide RMs are unfeasible, AT can potentially improve survival, encouraging clinicians to consider ATs in these high-risk groups. However, these findings are preliminary due to the small number of patients in subgroups. These results underscore the need for surgical precision to optimize RM width and tailored AT approaches based on RM status, improving risk stratification and treatment planning.

## Figures and Tables

**Table 1:** Patient characteristics.

Characteristics	Total (n = 139) No. (%)	Wide RM (n = 65) No. (%)	Close RM (n = 32) No. (%)	Positive RM (n = 42) No. (%)	*p*-Value
Age, years					0.073
<70	69 (49.6)	39 (60.0)	13 (40.6)	17 (40.5)	
≥70	70 (50.4)	26 (40.0)	19 (59.4)	25 (59.5)	
Sex					0.127
Male	84 (60.4)	45 (69.2)	16 (50.0)	23 (54.8)	
Female	55 (39.6)	20 (30.8)	16 (50.0)	19 (45.2)	
CEA, ng/mL					0.292
≤5	127 (91.4)	57 (87.7)	31 (96.9)	39 (92.9)	
>5	12 (8.6)	8 (12.3)	1 (3.1)	3 (7.1)	
CA 19-9, U/mL					0.079
≤37	32 (23.0)	13 (20.0)	12 (37.5)	7 (16.7)	
>37	107 (77.0)	52 (80.0)	20 (62.5)	35 (83.3)	
Surgical procedure					<0.001
PD	103 (74.1)	58 (89.2)	22 (68.8)	23 (54.8)	
Bile duct resection	36 (25.9)	7 (10.8)	10 (31.2)	19 (45.2)	
Tumor size, cm					0.095
<3	87 (62.6)	46 (70.8)	20 (62.5)	21 (50.0)	
≥3	52 (37.4)	19 (29.2)	12 (37.5)	21 (50.0)	
Histologic grade					0.180
WD/MD	115 (82.7)	56 (86.2)	23 (71.9)	36 (85.7)	
Poorly differentiated	24 (17.3)	9 (13.8)	9 (28.1)	6 (14.3)	
Portal-vein invasion					0.251
Absent	134 (96.4)	63 (96.9)	32 (100.0)	39 (92.9)	
Present	5 (3.6)	2 (3.1)	0 (0.0)	3 (7.1)	
Pancreatic invasion					0.268
Absent	79 (56.8)	33 (50.8)	18 (56.2)	28 (66.7)	
Present	60 (43.2)	32 (49.2)	14 (43.8)	14 (33.3)	
Duodenal invasion					0.337
Absent	121 (87.1)	54 (83.1)	28 (87.5)	39 (92.9)	
Present	18 (12.9)	11 (16.9)	4 (12.5)	3 (7.1)	
LVI					0.009
Absent	95 (68.3)	50 (76.9)	24 (75.0)	21 (50.0)	
Present	44 (31.7)	15 (23.1)	8 (25.0)	21 (50.0)	
PNI					0.230
Absent	48 (34.5)	27 (41.5)	8 (25.0)	13 (31.0)	
Present	91 (65.5)	38 (58.5)	24 (75.0)	29 (69.0)	
T stage					0.363
1	35 (25.2)	18 (27.7)	5 (15.6)	12 (28.6)	
2–3	104 (74.8)	47 (72.3)	27 (84.4)	30 (71.4)	
No. of dissected LN, mean ± SD	13.3 ± 9.3	15.1 ± 9.8	13.4 ± 10.0	10.4 ± 7.3	0.039
Adjuvant therapy					0.147
No	91 (65.5)	48 (73.8)	19 (59.4)	24 (57.1)	
Yes	48 (34.5)	17 (26.2)	13 (40.6)	18 (42.9)	

RM, resection margin; CEA, carcinoembryonic antigen; CA 19-9, carbohydrate antigen 19-9; PD, pancreaticoduodenectomy; WD, well differentiated; MD, moderately differentiated; LVI, lymphovascular invasion; PNI, perineural invasion; LN, lymph node; SD, standard deviation.

**Table 2:** Multivariate analysis of prognostic factors for LRC, PFS, and OS.

Variable	LRC		PFS		OS	
	HR (95% CI)	*p*-Value	HR (95% CI)	*p*-Value	HR (95% CI)	*p*-Value
CA 19-9 (≤37 vs. >37 U/mL)	3.164 (1.309–7.646)	0.011 *	2.265 (1.163–4.413)	0.016 *	-	*-*
Tumor size (<3 vs. ≥3 cm)	1.529 (0.872–2.680)	0.138	-	-	-	*-*
Grade (WD/MD vs. poorly)	-	-	0.619 (0.351–1.090)	0.097	-	*-*
LVI (absent vs. present)	-	-	-	-	2.319 (1.451–3.707)	<0.001 *
T stage (1 vs. 2–3)	1.039 (1.001–1.078)	0.044 *	1.023 (0.995–1.052)	0.107	-	*-*
RM status					-	*-*
Wide	Reference		Reference		-	*-*
Close	2.308 (1.120–4.757)	0.023 *	1.497 (0.795–2.819)	0.212	-	*-*
Positive	1.950 (1.004–3.787)	0.048 *	1.898 (1.109–3.247)	0.019 *	-	*-*

LRC, locoregional control; PFS, progression-free survival; OS, overall survival; HR, hazard ratio; CI, confidence interval; CA 19-9, carbohydrate antigen 19-9; WD, well differentiated; MD, moderately differentiated; LVI, lymphovascular invasion; RM, resection margin. * Statistically significant in the multivariate analysis.

## Data Availability

The datasets analyzed in this study are available from the corresponding author upon reasonable request.

## Abbreviations

The following abbreviations are used in this manuscript:

AT	Adjuvant therapy
CA 19-9	Carbohydrate antigen 19-9
CCC	Cholangiocarcinoma
CCRT	Concurrent chemoradiotherapy
CEA	Carcinoembryonic antigen
CI	Confidence interval
CT	Computed tomography
HR	Hazard ratio
LN	Lymph node
LRC	Locoregional control
LVI	Lymphovascular invasion
MD	Moderately differentiated
MRI	Magnetic resonance imaging
OR	Odds ratio
OS	Overall survival
PD	Pancreaticoduodenectomy
PFS	Progression-free survival
PNI	Perineural invasion
RM	Resection margin
RT	Radiotherapy
SD	Standard deviation
WD	Well differentiated
